# Complete Genome Sequences of an Avian Orthoreovirus Isolated from Guangxi, China

**DOI:** 10.1128/genomeA.00495-13

**Published:** 2013-07-11

**Authors:** Liqiong Teng, Zhixun Xie, Liji Xie, Jiabo Liu, Yaoshan Pang, Xianwen Deng, Zhiqin Xie, Qing Fan, Sisi Luo

**Affiliations:** Guangxi Key Laboratory of Animal Vaccines and Diagnostics, Guangxi Veterinary Research Institute, Nanning, Guangxi Province, China

## Abstract

We report the complete genomic sequences of an avian orthoreovirus, strain GuangxiR1, isolated from a chicken flock in Guangxi Province, southern China, in 2000. Phylogenetic analyses suggest that the strain is closely related to the S1133 strain, which is associated with tenosynovitis, but is far different from strain AVS-B, which is associated with runting-stunting syndrome in broilers.

## GENOME ANNOUNCEMENT

Avian orthoreovirus (ARV) is the etiological agent of viral arthritis, runting-stunting syndrome, and tenosynovitis in chickens, causing significant economic losses in the poultry industry (1, 2). Avian orthoreovirus belongs to the genus *Orthoreovirus*, family *Reoviridae* (3). The genome of 10 RNA segments was divided into three size classes, designated large (L1, L2, L3), medium (M1, M2, M3), and small (S1, S2, S3, and S4) (4). These segments encode 10 viral structural proteins (λA, λB, λC, μA, μB, μBC, μBN, σC, σA, and σB) and five nonstructural proteins (μNSC, μNS, σNS, P10, and P17) (5, 6, 7). The genetic characterization of the complete genomic sequence is limited to a few strains (8).

The avian orthoreovirus strain GuangxiR1, which caused paralysis and hock joints in commercial broilers, was isolated from Guangxi, southern China, in 2000 (9, 10). The nucleotide sequences of GuangxiR1 were amplified through reverse transcription PCR (RT-PCR). The amplified products were purified using a DNA purification kit (TaKaRa Biotechnology) and cloned into pMD18-T vector (TaKaRa, Dalian, China). The PCR product DNA sequencing was performed by two commercial DNA sequence service companies (Invitrogen, Biotechnology Co., Ltd. and Shenzhen Huada Genomics Co., Ltd.). Nucleotide sequence analysis and alignment were done using the MEGA v. 4.1 software package (MEGA Software).

The complete genome of GuangxiR1 is 23,494 bp. The full lengths of the L1, L2, L3, M1, M2, M3, S1, S2, S3, and S4 segments are 3,959, 3,830, 3,907, 2,283, 2,158, 1,996, 1,643, 1,324, 1,202, and 1,192 nucleotides, respectively. The deduced lengths of the structural and nonstructural proteins λa, λB, λC, μA, μB, μNS, σC, σA, σB, and σNS are 1,293, 1,259, 1,285, 732, 676, 635, 326, 416, 367, and 367 amino acids, respectively. The σC protein, involved in the induction of apoptosis, is coded by the S1 segment. Amino acid comparative analyses indicated that σC shares the highest sequence homology (98.2%) with that from the S1133 strain, associated with tenosynovitis (GenBank accession no. AF330703), but only 48.5% with that from the AVS-B strain, associated with runting-stunting syndrome in broilers (GenBank accession no. FR694197). These data are useful for analyses of the epidemiology and evolutionary characteristics of avian orthoreovirus GuangxiR1.

### Nucleotide sequence accession numbers.

The complete genome sequences of GuangxiR1 are available in GenBank. The accession no. KC183743 to KC183752 correspond to L1, L2, L3, M1, M2, M3, S1, S2, S3, and S4, respectively.
